# Sexual health and well-being across the life course: call for papers

**DOI:** 10.2471/BLT.23.291043

**Published:** 2023-12-01

**Authors:** Manjulaa Narasimhan, Kate Gilmore, Raul Murillo, Pascale Allotey

**Affiliations:** a Department of Sexual and Reproductive Health and Research, World Health Organization, 20 Avenue Appia, 1211 Geneva, Switzerland.; b Department of International Development, London School of Economics and Political Science, London, England.; c Centro Javeriano de Oncología, Hospital Universitario San Ignacio, Bogotá, Colombia.

Sexual health is fundamental to overall health and well-being, to the dignity of the individual and to the social and economic development of communities and countries.^1^ Three decades ago, participants of the international conference on population and development recognized sexual health as distinct from and broader than reproductive health. Sexual health is not limited to counselling and care related to reproduction and sexually transmitted diseases.^2^

The World Health Organization (WHO) working definition of sexual health is “a state of physical, emotional, mental and social well-being in relation to sexuality; it is not merely the absence of disease, dysfunction or infirmity.”^3^ Viewed affirmatively, sexual health is a positive and respectful approach to sexuality and sexual relationships, including “consensual and safe sexual experiences, free from coercion, discrimination, abuse or violence. For sexual health to be attained and maintained, the sexual rights of all persons must be respected, protected and fulfilled.”^3^

Promoting sexual health is among the five core aspects of the 2004 WHO global reproductive health strategy, endorsed by 191 Member States at the 57th World Health Assembly.^4^ In *Transforming our world: the 2030 Agenda for Sustainable Development,* target 3.7 aims to ensure universal access to sexual and reproductive health-care services as part of meeting the health-related sustainable development goal.^5^ Yet, despite Member State endorsement of these global initiatives, more than half of the world’s population continues to have limited or no access to sexual and reproductive health services over their lifetime.^6^

Furthermore, despite the universality of sex, cultural, religious, political and other forms of opposition to efforts to uphold sexual health and well-being continue to adversely affect progress at national, regional and global levels. The relative lack of public health support for sexual health has resulted in continued efforts to deny that there are universal human rights to sexual health and well-being.

Science drives WHO’s programme on sexual health.^1^ WHO’s record of evidence generation, synthesis and research shows how investing in science can operationalize human rights towards health impact. This programme is also firmly grounded in human rights as universal and non-exclusionary, as affirmed by the United Nations Charter and elaborated in the Universal Declaration of Human Rights.^7^

The *Bulletin of the World Health Organization* will publish a theme issue on sexual health and well-being across the life course that aims to create a dialogue on this topic, and highlight current evidence from both health system and people-centred perspectives. We are interested in inclusive and holistic approaches to the health of people in all their diversity and across their life course, of women from pre-menarche to after menopause; of underserved individuals (such as lesbian, gay, bisexual, transgender, queer or intersex) and communities (such as indigenous people); and of those for whom sexual activity is often seen as inappropriate (for example adolescents, older adults and people with disabilities). 

The theme issue will also highlight evidence-based and rights-based approaches that are applied to (i) specific intervention areas such as reproductive health, including sexually transmitted infections; (ii) contexts such as fragile and humanitarian settings; (iii) realizing agency and bodily autonomy; (iv) information, education and counselling about sex and sexuality, including sex-positive approaches; (v) overlooked sexual health conditions; (vi) sexual dysfunction and sexual pain disorders; (vii) transforming harmful norms, attitudes and taboos about sexuality; (viii) ensuring quality, ethical and voluntary sexual health services and care; and (ix) eliminating stigma, discrimination, coercion and violence. Special attention will also be given to societal and demographic changes, the implications of infectious diseases outbreaks and conflict, as well as the impact of innovations such as digital and self-care interventions.

The *Bulletin* welcomes contributions from all stakeholders including public health decision-makers, researchers, civil society and community representatives. Authors are encouraged from diverse regions, fields, ages, genders and communities. All papers considered for the theme issue need to provide an advance to the field. Submissions are due by 1 March 2024. Manuscripts should be submitted in accordance with the *Bulletin*’s guidelines for contributors (available at: https://www. who.int/publications/journals/bulletin/ contributors/guidelines-for-contributors) and the cover letter should mention this call for papers. Advancing sexual health and rights remains imperative to health for all. We look forward to bold, evidence-based insights. 

## References

[1] Sexual health. Geneva: World Health Organization; 2023. Available from: https://www.who.int/health-topics/sexual-health [cited 2023 Nov 3].

[2] International Conference on Population and Development Program of Action. New York: United Nations Department of Economic and Social Affairs; 1994. Available from: https://www.un.org/development/desa/pd/sites/www.un.org.development.desa.pd/files/files/documents/2020/Jan/un_1995_programme_of_action_adopted_at_the_international_conference_on_population_and_development_cairo_5-13_sept._1994.pdf [cited 2023 Nov 3].

[3] Defining sexual health: Report of a technical consultation on sexual health, 28–31 January 2002. Geneva: World Health Organization; 2006. Available from: https://www3.paho.org/hq/dmdocuments/2009/defining_sexual_health.pdf [cited 2023 Nov 3].

[4] Reproductive health strategy to accelerate progress towards the attainment of international development goals and targets. Geneva: World Health Organization; 2004. Available from: https://www.who.int/publications/i/item/WHO-RHR-04.8 [cited 2023 Nov 3]. 10.1016/s0968-8080(05)25166-2 16035592

[5] Sustainable development goal 3: ensure healthy lives and promote well-being for all at all ages. New York: United Nations Department of Economic and Social Affairs; 2015. Available from: https://sdgs.un.org/goals/goal3 [cited 2023 Nov 3].

[6] Starrs AM, Ezeh AC, Barker G, Basu A, Bertrand JT, Blum R, et al. Accelerate progress – sexual and reproductive health and rights for all: report of the Guttmacher*-*Lancet Commission. Lancet. 2018 Jun 30;391(10140):2642–92. 10.1016/S0140-6736(18)30293-9 29753597

[7] Universal declaration of human rights. Paris: United Nations General Assembly; 1948. Available from: https://www.un.org/en/about-us/universal-declaration-of-human-rights [cited 2023 Nov 3].

